# Assessment of Blood and Semen Detection and DNA Collection from Swabs up to Three Months after Deposition on Five Different Cloth Materials

**DOI:** 10.3390/ijms25063522

**Published:** 2024-03-20

**Authors:** Francisco Medina-Paz, Brandon Kuba, Emily Kryvorutsky, Gabriela Roca, Sara C. Zapico

**Affiliations:** 1 Department of Chemistry and Environmental Sciences, New Jersey Institute of Technology, Newark, NJ 07102, USA; fm368@njit.edu (F.M.-P.); blk4@njit.edu (B.K.); eck8@njit.edu (E.K.); 2 SERATEC mbH, 37079 Göttingen, Germany; gabriela.roca@seratec.com; 3 Anthropology Department, National Museum of Natural History, Smithsonian Institution, Washington, DC 20560, USA

**Keywords:** blood, semen, fabric, time, lateral flow immunochromatographic test, STR profiles

## Abstract

Body fluid identification plays a crucial role in criminal investigations. Because of their presence in many cases, blood and semen are the most relevant body fluids in forensic sciences. Based on antigen–antibody reactions binding unique proteins for each body fluid, serological assays represent one of the most rapid and highly specific tests for blood and semen. Currently, few studies have assessed the factors affecting body fluid identification by applying these assays. This work aimed to study the effect of different fabrics from clothes and time since deposition on identification through immunochromatographic tests for blood and semen, DNA isolation, and STR profiling from these samples. Body fluids were deposited on black- and white-dyed denim and cotton fabrics, and on leather. Afterward, blood and semen were sampled at 1 day, 30 days, and 90 days after deposition and identified by using the SERATEC^®^ HemDirect Hemoglobin Test and the PSA Semiquant and SERATEC^®^ BLOOD CS and SEMEN CS tests, respectively. Laboratory and crime scene tests presented similar performances for the detection of blood and semen stains on every tested fabric. No differences were found on band intensities between timepoints for all fabrics. It was possible to recover and identify blood and semen samples up to three months after deposition and to obtain full STR profiles from all the tested fabrics. Both body fluid STR profiles showed differences in their quality between 1 and 90 days after deposition for all fabrics except for black cotton for semen samples. Future research will expand the results, assessing body fluid identification on other substrates and under different environmental conditions.

## 1. Introduction

Body fluid identification at the crime scene has important repercussions on criminal investigations to characterize potential biological evidence and determine someone’s presence at the place. Body fluids of interest in forensic sciences include blood, semen, saliva, vaginal fluid, urine, and sweat [1].

Since the last century, a variety of methods have been developed and applied for the location and identification of relevant body fluids in crime scenes. The current testing methods for body fluid investigation rely on chemical, enzymatic, and/or serological assays. Chemical tests have been employed for several years and still play a critical role when referring to the location of an area of further forensic examination.

However, the poor specificity and sensitivity of these methods coupled with the destruction of the sample and/or inhibition of downstream processes (i.e., DNA profiling) limit their current application [2].

Serological assays are specific toward body fluid identification as they are based on antigen–antibody reactions binding unique or almost unique proteins for different body fluids such as peripheral and menstrual blood, semen, saliva, and urine. Within serological tests, the most common and quickest are the ones based on lateral flow immunochromatographic (LFI) tests. For blood, the commonly used serological tests are based on monoclonal anti-human hemoglobin antibodies that cross-react with primate blood [3]. In the case of semen, one of the most found body fluids at crime scenes, serological assays, are based on testing for prostate-specific antigen (PSA), a protein produced in considerable amounts only in the prostate [4]. Remarkably, PSA can be detected even in semen from azoospermic males as well as in contaminated or scarce samples such as laundered fabrics [5,6]. Some immunochromatographic tests use semenogelin, a protein that originates in the seminal vesicles and a substrate for the prostate-specific antigen (PSA) to identify seminal plasma in forensic samples [7,8].

Despite the widespread use of serological tests, there are few studies assessing factors affecting the identification of body fluids by applying LFI tests. Among these factors, there are two to consider when detecting body fluids at crime scenes: the substrate material on which the fluid has been deposited and the time since the sample was deposited on the substrate. There are numerous studies on the stability of body fluids and the effect of time and environmental conditions on it. Many chemical techniques have been developed since the early 1900s to study bloodstains’ stability and determine time since deposition [9]. In the late 20th century, more complex techniques to estimate time since the deposition of body fluids were introduced based on high-resolution liquid chromatography and diverse types of spectroscopies and spectrophotometry, as well as DNA/RNA-based techniques [10,11,12,13]. On the other hand, substrates can affect blood clotting and the bloodstain’s drying time [14]. Fabrics are a common type of substrate material on which body fluids can be encountered in forensic cases. Due to the inherent nature of body fluids, they spread and dilute when in contact with fabric [15]. Additionally, body fluid samples found at crime scenes are taken at best within hours, but they can even be taken months after the fluid has been deposited on the material. As mentioned before, efficient body fluid identification methods are characterized by their non-destructive nature. The main reason for these methods to be non-destructive is the preservation of DNA evidence. DNA profiling of the samples is the second step in a crime investigation after body fluid identification. Assuming the body fluid identification was carried out using LFI, DNA profiling could be carried out using the same swab used for the LFI or from another sample [16].

The present work assessed the effect of time and fabric type on body fluid identification over time from 1 day to 90 days after deposition of the fluid on denim, cotton, and leather fabrics and compared the performance of SERATEC^®^ HemDirect Hemoglobin and SERATEC^®^ PSA Semiquant (SERATEC^®^, Göttingen, Germany) to that of their crime scene (CS) counterparts. Additionally, this work assessed the human DNA recovery performance from sample swabs and evaluated the possibility of short tandem repeat (STR) profiling of samples extracted from different fabrics and after-deposition timepoints.

## 2. Results

### 2.1. Immunochromatographic Test Results

A band in the test line indicates the presence of the protein: hemoglobin or prostate-specific antigen (PSA); resulting in a positive immunochromatographic test. The band intensity was ranked according to the same scale used by the SERATEC^®^ company as an internal control (Supplementary Figure S1). Results from the laboratory and crime scene tests were evaluated separately as significant differences in the band intensities of semen samples between test types were found (*p* = 0.017). However, no differences were observed between the band intensities of laboratory and crime scene tests for blood samples (*p* = 0.15).

The Kruskal–Wallis tests showed a significant influence for both fabric (*p* = 9.003 × 10^−5^) and time (*p* = 0.030) variables over the band intensity of laboratory tests of blood samples, but only fabric (*p* = 0.013) showed influence over the band intensity of CS tests of the same body fluid. Regarding semen samples, no significant influence was observed either from fabric or from time over the band intensity of any test type (Supplementary Table S1).

**Figure 1:**
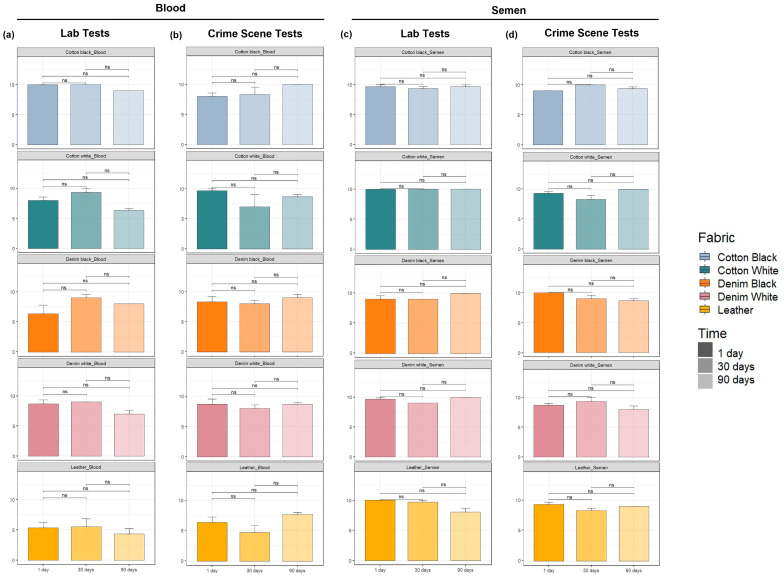
Average band intensity ranked values of immunochromatographic tests. (**a**) Blood laboratory tests; (**b**) blood crime scene tests; (**c**) semen laboratory tests; and (**d**) semen crime scene test samples. The label “ns” indicates a non-statistically significant difference among timepoints from each fabric type (Wilcoxon post hoc tests, *p* < 0.05, n = 3).

As shown in Figure 1, no significant differences were observed when comparing the band intensity of samples recovered from the same fabric over time either for laboratory or for crime scene tests of both body fluids studied in the present work. Overall, lower average band intensity values (G-values ≤ 6) for laboratory and CS tests of blood samples deposited on leather compared to the rest of the fabrics were also observed (Figure 1a,b). Remarkably, it was possible to obtain high average band intensity ranks (G-values > 6) for semen samples deposited on every evaluated fabric from 1 day to 90 days after deposition of the samples, both for laboratory and CS tests. A similar result was obtained for blood samples on all fabrics tested, except for leather, more evidently on laboratory tests.

### 2.2. Human DNA Quantification

The DNA concentration of the samples was assessed using Qubit. Human-specific DNA quantification was calculated using the PowerQuant System (Promega Corporation, Madison, WI, USA). The Kruskal–Wallis tests showed a significant effect for time (*p* = 0.0232) but not for fabric (*p* = 0.3985) on the human DNA concentrations of blood samples (Supplementary Figure S2a). A different trend was observed for semen samples in which an effect for fabric but not for time was found on human DNA concentrations (*p* = 0.0440 and *p* = 0.9658, respectively). An overall higher human DNA concentration (*p* = 0.0061) was also observed in semen compared to blood samples. Interestingly, a trend for lower human DNA concentrations was obtained from semen samples deposited on black-colored fabrics (cotton and denim) when compared to the white-colored fabrics (Supplementary Figure S2b).

### 2.3. DNA Profiling

It was possible to obtain a full DNA profile from all but one replicate corresponding to a blood sample deposited on black cotton fabric exposed for 1 day, where the resulting profile consisted of 26 out of 48 detected alleles. The quality of the profiles was assessed based on the calculations of two parameters as described by C. Zapico et al. (2022) [17]: total peak height (TPH) and peak height ratio (PHR). Blood and semen samples were analyzed separately. The Kruskal–Wallis tests revealed there was a significant influence of fabrics and timespans tested for this experiment over the TPH for both blood and semen samples (Supplementary Table S1).

**Figure 2:**
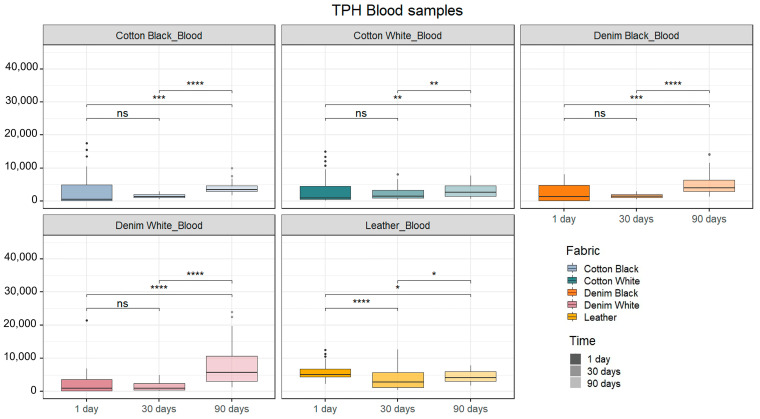
Average of total peak height (TPH) for blood samples. Total peak height is expressed as relative fluorescent units (RFUs). Asterisks indicate significant effects of time over band intensity for the same fabric and same body fluid (“****”: *p* ≤ 0.0001, “***”: *p* ≤ 0.001, “**”: *p* ≤ 0.01, “*”: *p* ≤ 0.05, “ns”: *p* > 0.05; Wilcoxon post hoc tests, n = 48).

Figure 2 represents the average TPH expressed as relative fluorescent units (RFUs) of the DNA profiles from blood and semen. For blood samples (Figure 2), we observed that 1-day and 30-day timespans tended to show lower peak heights compared to a 90-days timespan after deposition in all but leather-deposited samples. Blood samples deposited on leather showed higher TPH values at 1 day compared to 30 days after deposition. It is worth mentioning that no significant difference was observed between TPH values when comparing black and white cotton and black versus white denim (*p* = 0.7739 and *p* = 0.7968, respectively).

No differences over time were observed regarding the peak height ratio for black cotton and black denim fabrics or for leather. However, white cotton showed the highest PHR values at the 1-day timepoint, while white denim showed the highest PHR values at 1 day and 90 days compared to 30 days after deposition (Supplementary Figure S3).

**Figure 3:**
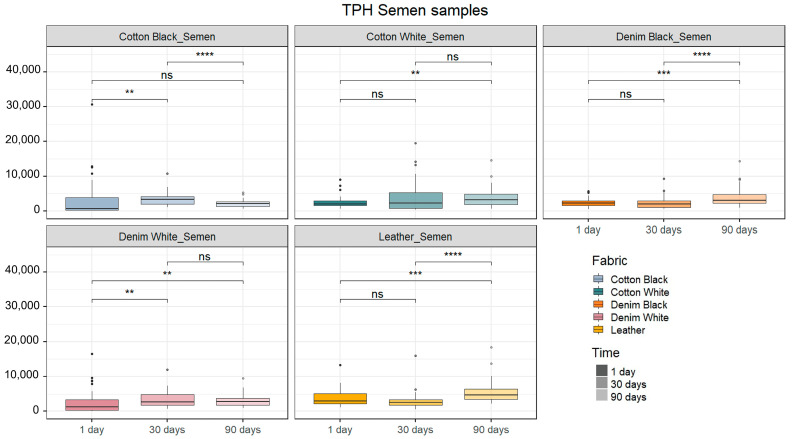
Average of total peak height (TPH) for semen samples. Total peak height is expressed as relative fluorescent units (RFUs). Asterisks indicate significant effects of time over band intensity for the same fabric and same body fluid (“****”: *p* ≤ 0.0001, “***”: *p* ≤ 0.001, “**”: *p* ≤ 0.01, “ns”: *p* > 0.05; Wilcoxon post hoc tests, n = 48).

On the other hand, significant differences in the TPH were observed between 1 day and 90 days after deposition for semen-stained white cotton fabrics. Moreover, semen samples deposited on black denim and leather showed higher TPH values at 90 days compared to 1 day and 30 days after deposition. Black cotton semen-stained fabrics showed lower TPH values at 1 day compared to 30 days, but showed no difference between 1 day and 90 days after deposition (Figure 3).

Additionally, significant differences were observed between the TPH values of semen samples deposited on cotton fabrics of distinct colors (black and white) but not between black and white denim fabrics (*p* = 0.04911 and *p* = 0.7968, respectively). Finally, the peak height ratio (PHR) calculations for semen presented similar values and no statistically significant differences were found between either fabrics or timespans (Supplementary Figure S4). An example of STR profile is showed in Supplementary Figure S5. 

## 3. Discussion

The main objective of the present work was to evaluate the impact of the time and the type of cloth after the deposition of blood and semen on the detection of these fluids, in the context of forensic analyses. To achieve this, the human DNA concentration and the band intensity of LFI tests and STR profiles of each body fluid were evaluated after exposure for 1 day, 30 days, and up to 90 days to an indoor environment and deposition on five different types of fabrics.

According to our results, neither time nor type of fabric showed a significant influence over the concentration of genetic material extracted from both blood and seminal fluid samples. It is worth mentioning that the blood DNA concentrations were notably lower than those in the semen samples. The concentrations of both body fluids matched with previously reported results of DNA extracted from cotton fabric stained with blood and semen. Fujii et al. [18] reported average nuclear DNA concentrations isolated from fresh bloodstains on cotton and denim of 0.53 ng/μL and 0.45 ng/μL, respectively. The same authors reported an average DNA concentration of around 11 ng/μL from fresh semen stains on cotton, while Davis et al. [19] reported an average concentration of 1.02 ng/μL for the same body fluid under similar conditions and deposition material.

Regarding the LFI band intensities, as described in the results section and supported by statistical analyses, laboratory and crime scene tests proved to be slightly but still significantly different regarding band intensity results but consistent with the conclusions. The differences in band intensity results between laboratory and CS tests might be explained by the differences in the thickness and size of the membranes between both test types. According to the manufacturer, the SERATEC^®^ HemDirect Hemoglobin Test has a sensitivity cutoff at 20 ng/mL. Thus, the appearance of a band in the HemDirect Test indicates a concentration ≥ 20 ng/mL of hemoglobin in the sample. For the SERATEC^®^ PSA Semiquant Test, the cutoff is set at 0.5 ng/mL of PSA, while, for the SERATEC^®^ PSA Semiquant CS Test, it is set at 2 ng/mL. Furthermore, the SERATEC^®^ PSA Semiquant Test and the PSA Semiquant CS count with an additional internal standard band whose band intensity correlates with a concentration of 4 ng/mL PSA. Band intensities the same as the internal standard band indicate values higher than 4 ng/mL, and dimmer bands indicate concentrations ≥ 0.5 ng/mL.

Proteins and DNA are molecules of different sizes; their diffusion and stability under dry conditions is different. However, for purposes with a forensic significance, a comparison has been performed to validate the collection of samples used for the identification of the type of body fluid and the construction of “a donor” profile. No relation was observed between human DNA quantification results and LFI band intensities.

The main goal of the LFI band intensity measurement was to evaluate the degradation of hemoglobin and PSA proteins in blood and semen, respectively, after the fluids were deposited on different textiles and exposed for up to 3 months to the environment. Then, it was possible to detect blood and semen up to 3 months after deposition of the samples although with variable but not significantly different band intensities. This is important to know when the amount of body fluid is too low. In our case we used 20 μL, when a normal drop of blood is approx. 35 μL [20]. From this amount of material, the quality of the human DNA obtained from all samples was further evaluated by examining the STR profiles. Full STR profiles were obtained from all blood and semen samples, except for one replicate of a 1-day-old black cotton blood-stained sample. This sample had a partial STR profile that might be attributed to an error when sampling with the swab, causing a low amount of DNA to be captured and consequently a very low STR signal, indistinguishable from background noise, when performing fragment analysis. The variability of profiles might also be attributed to the DNA extraction method itself, as some inhibitors (e.g., hematin) could have been isolated together with the nucleic acids at the time of the extraction [21,22]. However, for DNA extraction, we used silica columns that trap, clean, and finally elute the DNA, by getting rid of most, if not all, the possible inhibitors in the original sample; this method has been successfully used in other works [23].

Our results on blood detection by LFI agree with those of Misencik and Laux [3], who reported positive results (visible bands on tests) for evaluated blood samples deposited on different substrate materials and exposed to environmental conditions during a 28-day period. However, no direct comparison could be made to the results reported by Misencik and Laux as no intensity rank but only the presence/absence of the band was evaluated. It is worth mentioning that we were able to observe bands, fainter or brighter, in samples up to 90 days after deposition, meaning that the presence of hemoglobin was detected by the tests in bigger or smaller amounts. However, the band intensity variations between samples from different timepoints and fabrics were not statistically significant. Particularly, blood-stained samples on leather measured using both laboratory and CS tests showed the lowest band intensities compared to the rest of the fabrics. It is tempting to speculate that the lower band intensities from a leather substrate might have to do with the physicochemical properties of blood and leather, which make the extraction of the fluid more difficult compared to the rest of the tested substrates. Given the large variety of leather types and its worldwide application for seat and clothing manufacturing, the present results open a window into further research of this material under the forensic scope.

Regarding DNA quality, it has been reported that full STR profiles can be obtained from aged bloodstains from samples as old as 2 years and even from 8-year-old samples, although the peaks at STR loci with large amplicons > 250 bp tended to be lower or disappear [24]. Hanson and Ballantyne [25] reported positive LFI results and full autosomal STR profiles for human blood samples obtained from a set of bloodstains exposed to 22 °C and 50% humidity for up to 1 week from eleven individuals. This coincides with the results shown in the present work since it was possible to obtain complete profiles in samples up to 90 days (about 3 months) old. The stability of bloodstains over time under different environmental conditions such as temperature and humidity has been analyzed by diverse methodologies and reported in the literature. Even though the decomposition process of blood once outside the body has been largely studied and distinguishable chemical compounds have been found and characterized between “recent” and “older” bloodstains [9,12], it has also been shown that nucleic acids such as DNA and different kinds of RNA remain stable and whole enough to be used for downstream analyses over time lapses as long as 30 days after deposition and exposition of the samples to harsh conditions such as elevated temperatures, high humidity, and laundry cycles [26,27].

The semen samples’ results are also consistent with those reported by Twanabasu [28], who demonstrated that LFI performed correctly for semen-stained fabric samples up to 120 days after deposition at room temperature under sunlight. Srettabunjong, Betset [29] showed that the PSA concentration declined but was still detectable through ELISA assays up to 7 days after storage at room temperature. The latter observation agreed with a previous report by Jimenez-Verdejo, Osuna [30] quantifying PSA concentration from vasectomized and non-vasectomized volunteers, by solid-phase sandwich-type immune-enzymatic analyses. Even though previous studies reported no detectable PSA concentration after 7-day storage at room temperature, these discrepancies might be due to the different sensitivities of the performed assays, as the studies in which ELISA assays were performed reported minimum concentrations around 1000 ng/μL 1 week after storage while the sensitivity range for an LFI assay goes as low as 2 ng/μL [31]. Therefore, low concentrations after 7 days could be overlooked when ELISA assays were performed.

## 4. Materials and Methods

### 4.1. Sample Preparation

Blood from an African-American woman was purchased from the American Blood Bank Corporation (Miami, FL, USA). Semen from a Caucasian male was purchased from Lee Biosolutions (Maryland Heights, MO, USA). The New Jersey Institute of Technology Institutional Review Board (IRB) approved the procedures related to human body fluid experimentation (protocol number: 2110013076). Five types of fabrics were used for this experiment: denim (dyed black and white), cotton (dyed black and white), and leather (natural leather). Cotton swabs (cotton-tipped applicators with a sterile wood shaft; SARSTEDT, Nümbrecht, Germany) were used to take both blood and semen samples.

### 4.2. Experimental Design

Twenty microliters of blood and the same volume of semen were deposited on three different fabrics: denim (dyed black and white), cotton (dyed black and white), and natural leather. After 1 day, 30 days, and 90 days at room temperature, the samples were recovered using cotton swabs. Sampling for both body fluids and for the three timepoints was performed in triplicate. The samples were left under a Biosafety Cabinet Class II A2 model 1353 (Thermo Fisher Scientific, Waltham, MA, USA) at room temperature (20 °C), between 30 and 50% humidity, under a constant airflow at 973 m^3^/h. The swab was moistened in the extraction buffer provided with the SERATEC^®^ HemDirect Hemoglobin Test and the SERATEC^®^ PSA Semiquant Test for blood and semen, respectively (SERATEC^®^, Göttingen, Germany). Then, the swab was applied to the sample in a circular motion for approximately 30 s. The swabs were incubated under agitation in 300 μL of extraction buffer for 10 min. Afterward, three drops of the resulting solution were added to the immunochromatographic tests, and the results were recorded. The whole experiment described above was performed in duplicate: three replicates were carried out using SERATEC^®^ HemDirect Hemoglobin and SERATEC^®^ PSA Semiquant (for understanding, here, called laboratory tests) tests, and three other replicates were performed using SERATEC^®^ BLOOD and SERATEC^®^ SEMEN CS (CS: crime scene) tests. In total, 45 blood samples were tested with the SERATEC^®^ HemDirect Hemoglobin tests, and 45 blood samples were tested with the SERATEC^®^ BLOOD CS tests. Furthermore, 45 semen samples were tested with the SERATEC^®^ PSA Semiquant tests, and 45 semen samples were tested with the SERATEC^®^ SEMEN CS. Negative controls were carried out by swabbing, with each test type, a different part of the fabric where no sample was deposited. Positive controls were performed by adding the body fluid directly on the test. The band intensity scale shown in Supplementary Figure S1 was used to assess the results of the immunochromatographic tests. This scale was used to compare the samples among themselves and not to quantify them. The above-mentioned scale is currently used by the SERATEC^®^ company as an internal control to assess the tests results [17].

### 4.3. DNA Extraction

The total DNA was isolated from the swab of all samples, including positive and negative controls on the SERATEC^®^ extraction buffer using a modification of the DNeasy Blood and Tissue Kit (Qiagen^®^, Venlo, The Netherlands). As part of the buffer volume from the previous test was used in test performance, an additional volume of SERATEC^®^ HemDirect Hemoglobin or SERATEC^®^ PSA Semiquant test extraction buffer was added to the Eppendorf tube, up to 400 μL. While the swab was still inside the Eppendorf tube, 20 μL of proteinase K and 400 μL of AL buffer were added. The resulting mixture was vortexed for 15 s and incubated under agitation at 56 °C for 10 min. Then, 400 μL of ethanol was added and vortexed for 15 s. Finally, the mixture was transferred to the column following the manufacturer’s protocol. The DNA was recovered eluting in a final volume of 50 μL of AE buffer.

### 4.4. Total and Human DNA Quantification

Total DNA quantification was performed using the Qubit dsDNA Assay Kit (Life Technologies, Carlsbad, CA, USA) along with the Qubit Fluorometer 3.0, according to the manufacturer’s protocol and a previously published protocol [32]. Human DNA quantification was carried out using the PowerQuant System (Promega Corporation, Madison, WI, USA) along with the QuantStudio (Thermo Fisher Scientific, Waltham, MA, USA), according to the manufacturer’s protocol.

### 4.5. Nuclear DNA Profiling

The Promega PowerPlex^®^ Fusion 6C System (Promega Corporation, Madison, WI, USA) was used to amplify and characterize 23 autosomal STRs, 3 YSTRs, and the Amelogenin gene in 1 ng of DNA, based on Qubit quantification, according to the manufacturer’s protocol. Fragment analysis was carried out on SeqStudio (Thermo Fisher Scientific, Waltham, MA, USA). The fragment analysis was performed under the following parameters: 7 s injection time; 1200 volts injection voltage; 1440 s run time; and 9000 volts run voltage. DNA profiling was achieved through the Microsatellite Analysis software on Thermo Fisher Cloud (https://www.thermofisher.com/rs/en/home/digital-science/thermo-fisher-connect.html, accessed on 16 January 2024). A threshold of 150 RFUs for peak detection/analysis was used. Two different parameters were calculated to assess the quality of the DNA as described by [17]: total peak height (TPH), the total sum of the height of the peaks in a profile, and peak height ratio (PHR), the height of the smaller peak in a heterozygote pair divided by the height of the larger peak.

### 4.6. Statistical Analysis

Statistical analyses were carried out using custom R scripts. All data were analyzed using R version 4.2.2 [33] and R Studio version 2023.06.0+421 [34]. Then, data from each fabric type were analyzed individually using non-parametric Kruskal–Wallis tests and Wilcoxon post hoc comparison tests. Within the same fabric type, differences between timepoints were tested for significance using Wilcoxon pairwise comparison against the control group.

## 5. Conclusions

In conclusion, the findings presented in this study indicate that it is possible to recover and identify blood and semen samples up to three months after deposition, as well as to obtain full STR profiles from five common types of fabrics from clothes. No significant differences in band intensities were found between timepoints when analyzing samples individually by fabric type, either for laboratory or for crime scene tests. From another part, the blood stains samples’ STR profiles did show differences in quality when comparing samples 1 day to 90 days after the deposition profiles for all fabrics. The same results were obtained for semen stains except for the black cotton fabric. The present work deepens the knowledge about the reliable application of routinely used tools for crimes discovered weeks after been committed. Future research will be able to expand the results, assessing the detection of body fluids in other fabrics and different environmental conditions.

## Data Availability

The data presented in this study are available upon request from the corresponding author. The data are not publicly available due to privacy issues.

## Supplementary Materials

The following supporting information can be downloaded at https://www.mdpi.com/article/10.3390/ijms25063522/s1.
