# Supplementary material for: Assessment of Blood and Semen Detection and DNA Collection from Swabs up to Three Months after Deposition on Five Different Cloth Materials
Source: Int J Mol Sci. 2024 Mar 20;25(6):3522. doi: 10.3390/ijms25063522 (PMC10971324; doi:10.3390/ijms25063522)

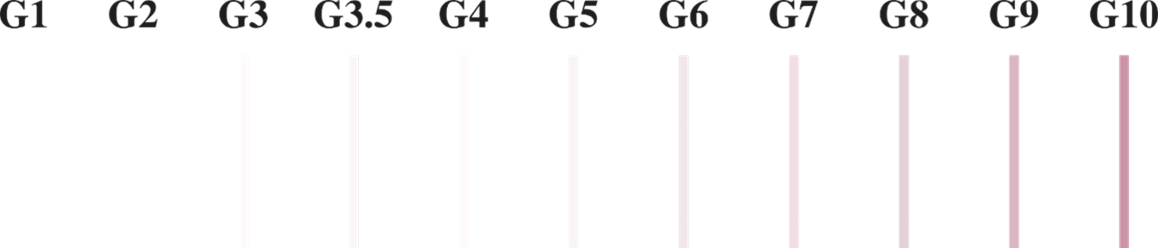


**Supplementary Figure S1.** Band intensity scale used by SERATEC® to assign ranks to the immunochromatographic tests signal


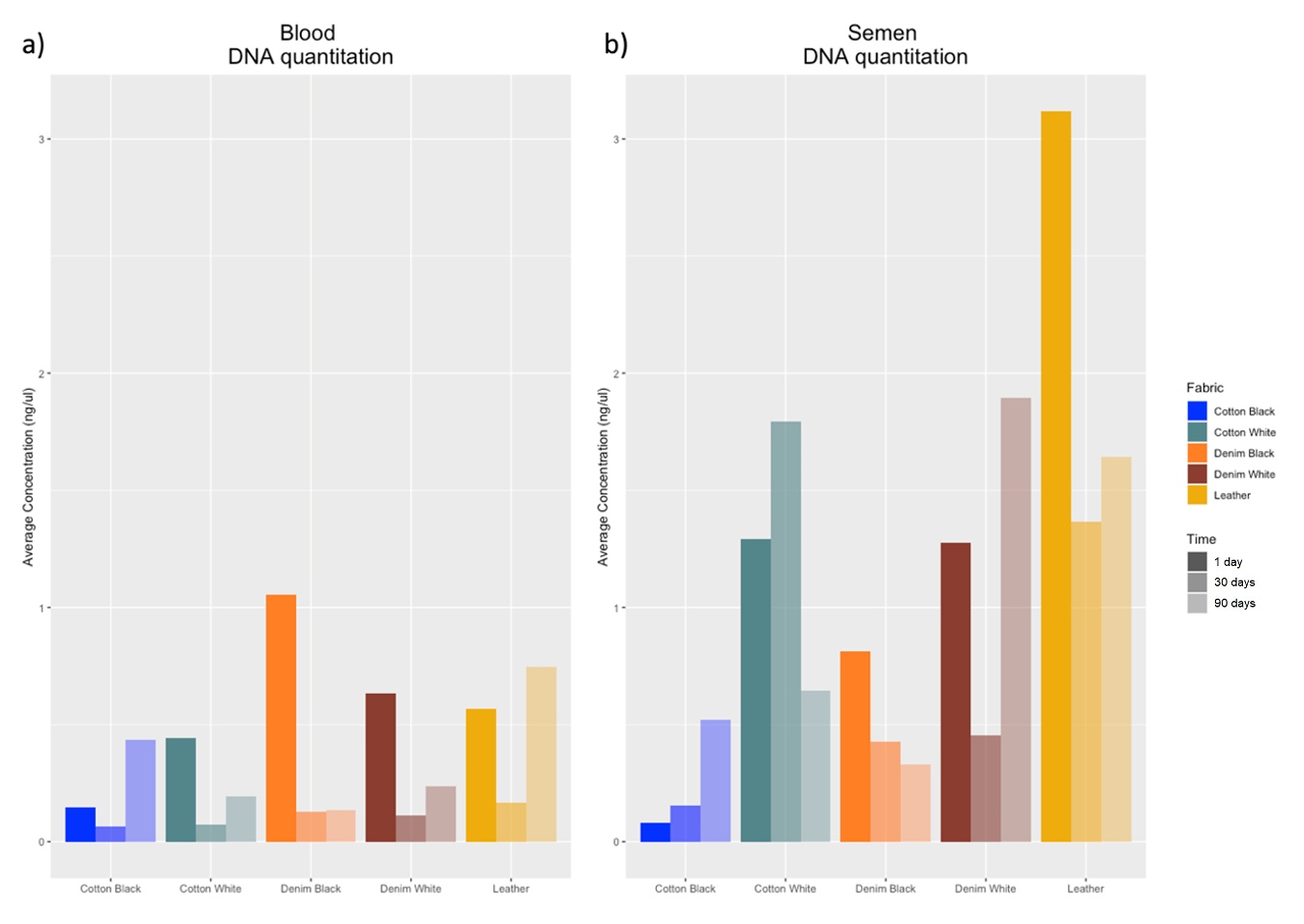


**Supplementary Figure S2.** Human DNA quantification. Average of human DNA concentration measured using the PowerQuant System from (a) blood and (b) semen samples


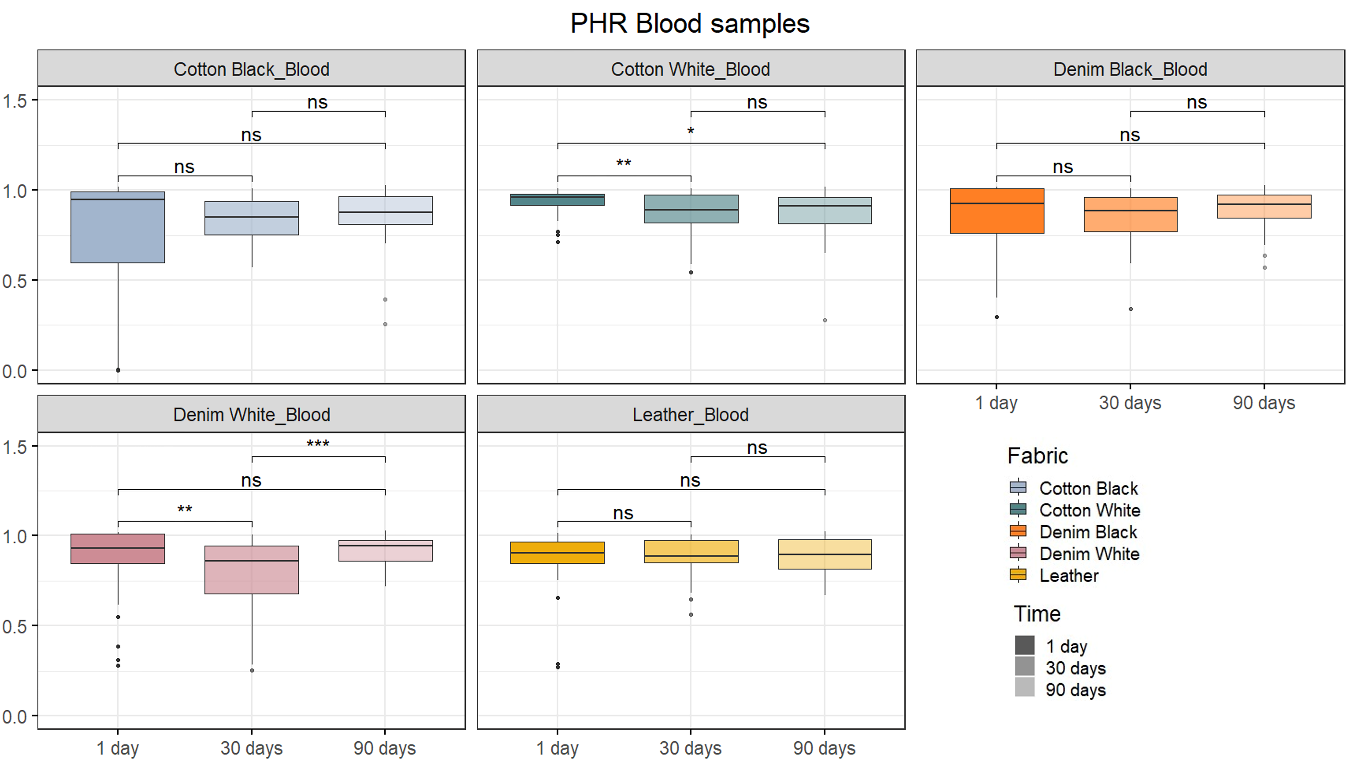


**Supplementary Figure S3.** Average of peak height ratio (PHR) of the DNA profiles for semen samples (“***”: p ≤ 0.001, “**”: p ≤ 0.01, “*”: p ≤ 0.05, “ns”: p > 0.05; Wilcoxon post hoc tests, n = 48).


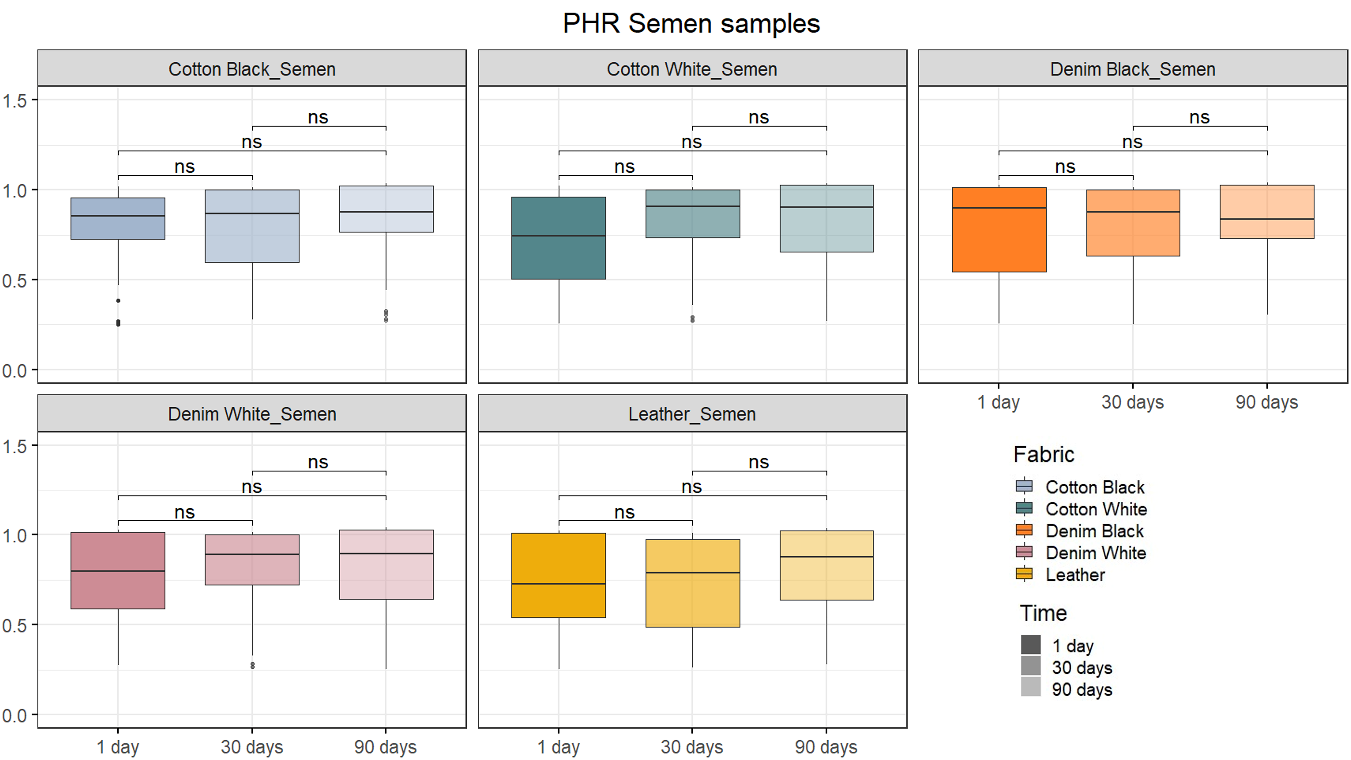


**Supplementary Figure S4.** Average of peak height ratio (PHR) of the DNA profiles for semen samples (“ns”: p > 0.05; Wilcoxon post hoc tests, n = 48).

**Supplementary Figure S5.** Representative STR profiles from samples extracted from different clothing and deposition times. a) Leather 3 months blood samples; b) Leather semen 3 months samples.


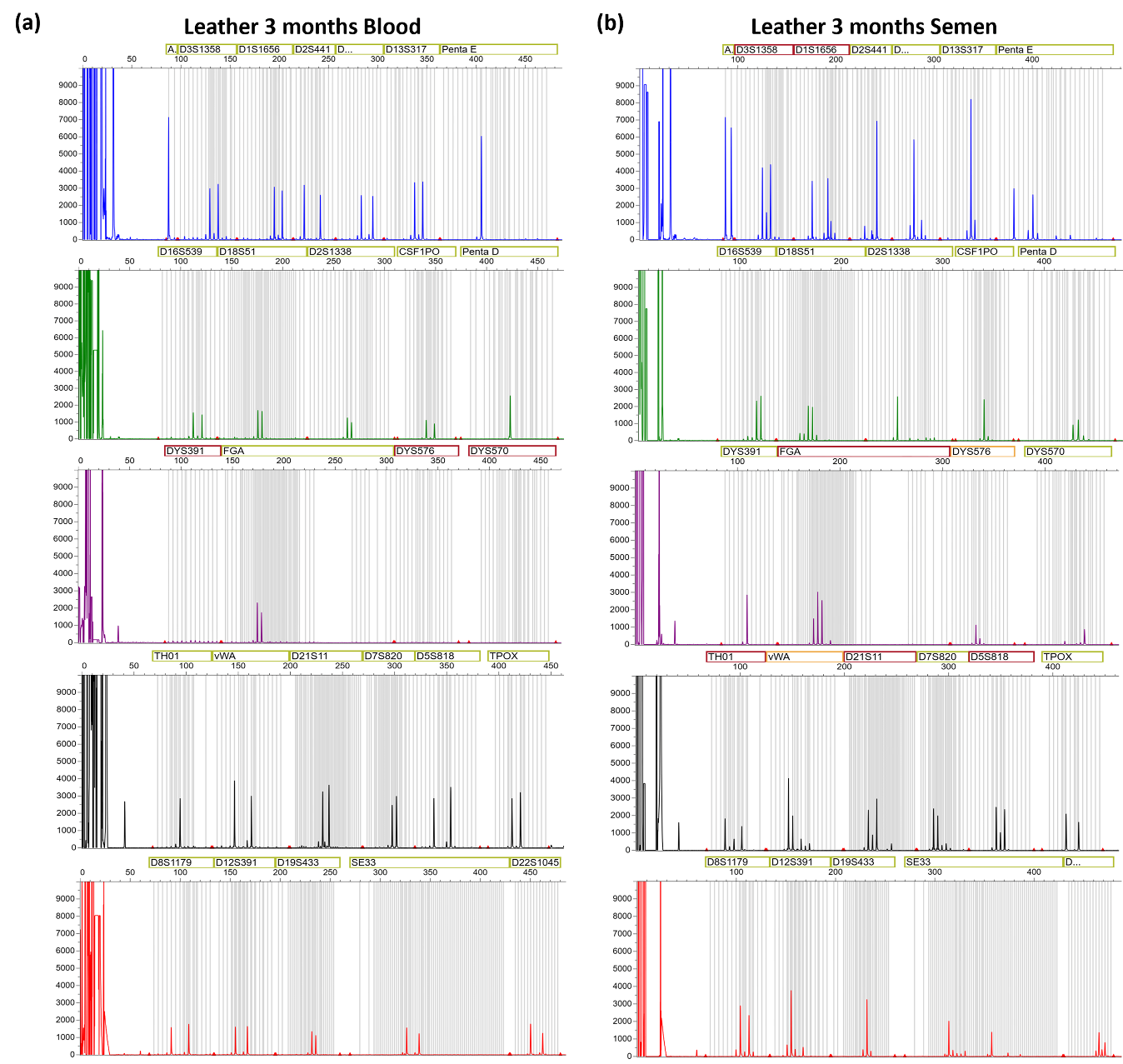

Supplement: Supplementary file 1 [file ijms-25-03522-s001.zip › Supplementary Figures.docx]
